# Variability in diagnostic and therapeutic decision-making for endodontic-periodontal lesions: evidence from a cross-sectional study

**DOI:** 10.3389/fpubh.2026.1795184

**Published:** 2026-04-30

**Authors:** Yasir Dilshad Siddiqui, Malik Zain Ul Abideen, Muhammad Rizwan Memon, Farah Tahir, Ammar Ahmed Siddiqui, Muhammad Nadeem Baig, Mohammed Katib Alrowili, Azhar Iqbal, Raha Ahmed, Ahmad Salaar

**Affiliations:** 1 Department of Preventive Dentistry, College of Dentistry, Jouf University, Sakaka, Saudi Arabia; 2 Department of Dental Education and Research, Bakhtawar Amin Medical and Dental College, Multan, Pakistan; 3 Department of Prosthetic Dental Sciences, College of Dentistry, Jouf University, Sakaka, Saudi Arabia; 4 Department of Pharmacy, Abasyn University, Islamabad, Pakistan; 5 Department of Preventive Dental Sciences, College of Dentistry, University of Hail, Hail, Saudi Arabia; 6 Department of Restorative Dentistry, College of Dentistry, Jouf University, Sakaka, Saudi Arabia; 7 College of Dentistry, Jouf University, Sakaka, Saudi Arabia; 8 Afghanistan Dental Association, Kabul, Afghanistan

**Keywords:** endodontic-periodontal lesions, diagnosis, treatment, dental practitioners, knowledge

## Abstract

**Background:**

The endodontic-periodontal lesions (EPLs) are a complex clinical condition whose diagnosis, treatment, and prognosis remain challenging. Therefore, a clear understanding of appropriate diagnostic and therapeutic approaches is essential for optimal treatment planning. This study aimed to evaluate dental practitioners' knowledge of diagnostic and therapeutic strategies for EPLs in Pakistan.

**Methods:**

A cross-sectional survey was conducted among 380 registered dental practitioners in Pakistan using a self-administered questionnaire. Participants included 134 females (35.3%) and 246 males (64.7%), with the majority aged 25**–**30 years. The questionnaire assessed diagnostic, therapeutic, and prognostic knowledge of EPLs. Data were analyzed using descriptive statistics, Pearson's chi-square test, Fisher's exact test, and binary logistic regression.

**Results:**

There was a substantial range in the knowledge among participants based on their demographic characteristics and profession. Unadjusted analysis showed that a statistically significant association existed between knowledge levels and independent variables, including age (*p* < 0.001), professional experience (*p* < 0.001), specialization (*p* < 0.001), and continuing professional training (*p* < 0.001); however, there was no statistically significant association found between organization type (*p* = 0.076). Multivariate logistic regression analysis indicated that practitioners aged 31–35 had a greater odds ratio [adjusted odds ratio (AOR) = 1.95, 95% confidence interval (CI): 0.90–4.22; *p* = 0.091] of good knowledge compared to those aged 25–30. However, practitioners aged 36–40 were significantly more likely to have good knowledge (AOR = 2.99, 95% CI: 1.38 – 6.47; *p* = 0.005).

**Conclusion:**

Practitioners had better therapeutic knowledge than diagnostic knowledge of EPLs, which indicates an overall above-average degree of awareness of EPLs, with noted gaps in diagnostic accuracy. Focused educational initiatives and evidence-based clinical practice guidelines may help improve practitioner decision-making for diagnosing EPLs as well as optimize the care provided for patients with EPLs, leading to improved patient outcomes.

## Introduction

**Figure 1:**
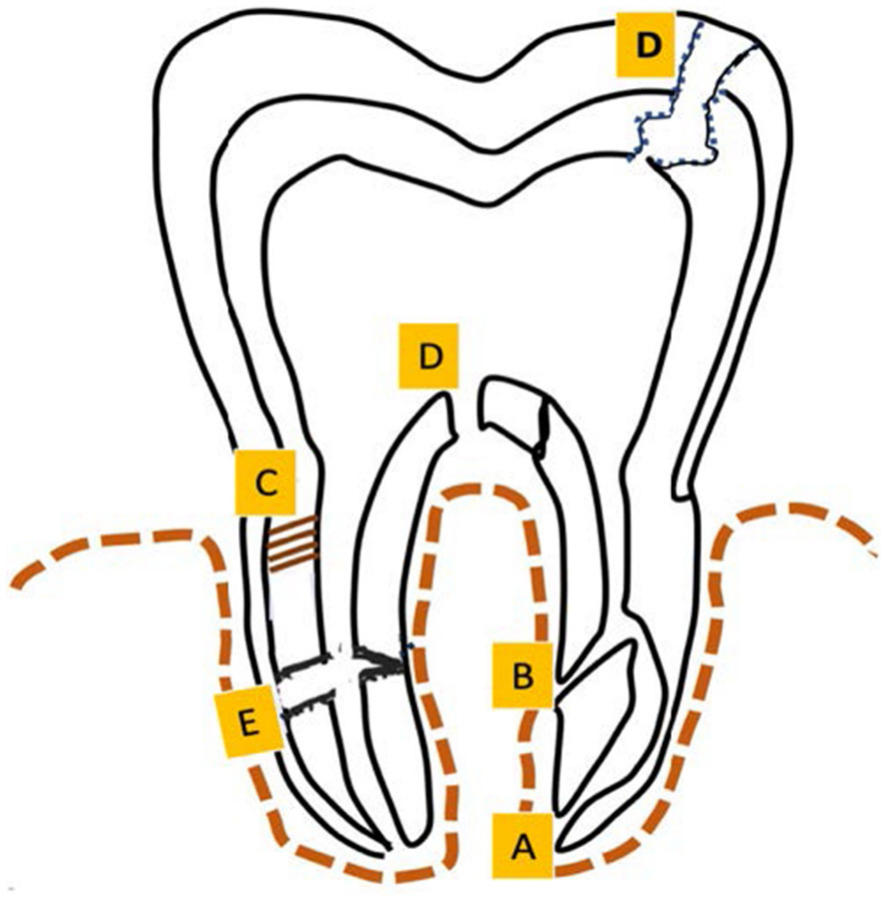
Pathways of communication between pulp and periodontium. **(A)** Apical foramen: This foramen can vary in number per tooth and serves as an entry and exit point for bacteria. **(B)** Lateral canals: These canals can be present along any part of the root surface, including the furcation area. **(C)** Dentinal tubules: The odontoblastic processes extend from the dentin–pulp complex to the root surfaces through the dentinal tubules, establishing communication when the root surface cementum is compromised. **(D)** Perforations: Perforations can occur due to resorption, caries, or iatrogenic damage, and the prognosis for the tooth depends on the extent and location of the perforation. **(E)** Fractures: Horizontal fractures are managed depending on their location, whereas vertical fractures usually render the tooth non-restorable.

Periodontium and endodontium are two anatomically and physiologically connected components that allow diseases in one location to potentially affect the other (1, 2), resulting in the formation of endodontic–periodontal lesions (EPLs) or combined lesions (CLs). Owing to the overlap of clinical signs and symptoms, the diagnosis of both types of lesions can be difficult (3, 4). An accurate understanding of the relationship between the periodontium and the dental pulp is necessary for a correct diagnosis (3, 4). Although the periodontium and the dental pulp are anatomically and functionally distinct tissues, multiple pathways enable biological communication between them, permitting the exchange of microorganisms and inflammatory mediators. These pathways include the apical foramen, exposed dentinal tubules, lateral and accessory canals, specific anatomical variations, and pathological conditions such as root fractures and perforations (3) (Figure 1).

The success of treatment planning and prognosis for EPLs depends on the practitioner's ability to correctly determine the origin of the lesion (5, 6). For example, if the initial pathology is caused by primary endodontic disease or secondary periodontal disease, the initial course of treatment should be endodontic therapy, with an evaluation at two to three months to determine whether periodontal treatment is needed. The extent of periodontal involvement has a direct effect on prognosis (3). Simultaneous endodontic and periodontal treatments have also been recognized as valid treatment options for managing EPLs (3, 7). Several diagnostic procedures are required to allow an accurate diagnosis of EPLs, including a detailed history, a careful examination of both soft and hard tissues of the oral cavity, pulp testing, and thorough periodontal probing (8). Identifying the cause of the pathology is also essential in developing an effective treatment plan (3).

Although several classification systems for EPLs have been proposed, including those by Al-Fouzan (3) and Rotstein (9), these conditions remain challenging for clinicians to diagnose, treat, and predict. The 2017 World Workshop classification (published in 2018; AAP/EFP), including the endodontic–periodontal lesion framework, provides the current overarching framework for periodontal diseases, including EPLs (10, 11).

However, despite these developments, variability in classification approaches and terminology continues to contribute to diagnostic complexity in clinical practice. In addition, the clinical and radiographic characteristics of EPLs can be misleading, potentially resulting in inaccurate diagnosis and inappropriate treatment decisions, such as premature tooth extraction (3, 12). Therefore, EPLs have a significant impact across multiple dental disciplines, and a multidisciplinary approach is needed to accurately diagnose and manage these conditions effectively (9).

Over the last few decades, the provision of dental care in Pakistan has progressed significantly; however, the content of dental education has not always been in line with modern scientific and clinical advances. Dental curricula generally have limited integration of evidence-based practice for the management of complex conditions such as EPLs (13). Although several international studies have investigated the knowledge and clinical practices of dental practitioners related to EPLs (14), national-level evidence from Pakistan remains limited. Therefore, to better understand how dental practitioners in Pakistan diagnose and manage EPLs, the present study was designed to evaluate diagnostic, therapeutic, and prognostic approaches and identify demographic and professional predictors of knowledge variability. The findings are intended to inform improvements in dental education, continuing professional development, and standardized clinical guidelines. We hypothesized that practitioners with greater clinical experience, postgraduate qualifications, and engagement in continuing professional education would demonstrate higher levels of diagnostic and therapeutic knowledge of EPLs.

## Materials and methods

### Study design and setting

The study received approval from the Research Ethics Committee of the Bakhtawar Amin Medical & Dental College, Pakistan (Reference No. 1189/23/COD). This was an observational study with a descriptive cross-sectional design conducted among registered dental practitioners in Pakistan, regardless of their gender or area of practice. Data collection took place from January 2024 to March 2024. All participants were informed about the purpose of the study and assured that their participation was voluntary. Electronic informed consent was obtained. Identifiable data were only used for quality control during data cleaning and were excluded from all subsequent analyses to maintain confidentiality and anonymity.

### Participants and sampling strategy

Data were collected via an online Google Form, and in view of the logistical challenges associated with recruiting a large sample of dental practitioners across Pakistan, a non-probability snowball sampling method was adopted. The data collection instrument was a self-completion questionnaire, and it was disseminated to participants through a variety of digital media, including WhatsApp, Facebook, and email. The participants were also encouraged to share the link to the questionnaire with colleagues working in dentistry, and consequently, the snowball sampling method resulted in a final sample size of 380. Participants were included if they were registered dental practitioners currently practicing in Pakistan, held at least a BDS or equivalent qualification, agreed to provide informed consent, and completed the questionnaire. Exclusion criteria included dental students, non-active practitioners, incomplete surveys, and duplicate entries on the basis of a review of the timestamp and metadata.

### Sample size and data quality control

To eliminate duplicate submissions of the questionnaire, all submissions were checked against submission timestamps, and where voluntary identifiers such as email addresses or names had been provided by the respondent, these were cross-checked for duplicate submissions. If duplicate submissions were identified, only the initial complete submission was retained. The survey was closed in March after five consecutive days with no new responses, which was used as an operational criterion to end recruitment. Using the Raosoft Calculator, which is based on a response distribution of 50%, a 95% confidence level, and a margin of error of 5%, a minimum sample size of 385 participants was calculated. In total, 391 responses to the questionnaire were received, but 11 responses were omitted from the analysis because they were incomplete or duplicated; consequently, a total of 380 responses were analyzed. Although the final sample size was marginally below the calculated minimum, this shortfall (< 2%) is unlikely to meaningfully affect the statistical precision of the primary analyses. Nonetheless, findings from subgroup analyses, particularly those involving categories with small cell sizes, should be interpreted with caution.

### Data collection instrument

The current study utilized a self-administered questionnaire adapted from a previous study (14). A few minor alterations were made to enhance readability, contextual relevance, and linguistic clarity for dentists practicing in Pakistan. The rest of the items from the original instrument, including its format, response options, and assessment methods, remained the same. No modifications were made to the way questions are formatted (i.e., yes/no), nor were there any adjustments to how scores are calculated, which remained consistent with the authors' original study. Since the instrument was developed prior to the 2017 classification, when the AAP/EFP published their new classification criteria, it reflects older definitions of disease states that do not include some of the details of the most recent classifications. To ensure that the questionnaire reflected the regional dental care setting in Pakistan, it was reviewed by a panel of experts familiar with the dental healthcare delivery systems in Pakistan. Modifications were made to the questionnaire to reflect regional terminology and practices. Additionally, several of the questions were rephrased to make them easier to understand and to accommodate practitioners with varying degrees of proficiency. Following expert review, the questionnaire was pilot tested with a small group of local dental practitioners to verify its clarity and applicability. Minor revisions were made to the questionnaire on the basis of feedback from the pilot test to increase the clarity of the questions.

### Validity and reliability

The content validity was determined by having a panel of eight endodontic and periodontal experts assess the questionnaire for relevance, clarity, and suitability for measuring the practitioners' level of knowledge regarding EPLs. The internal consistency was determined by calculating a Cronbach's alpha of 0.85, which is indicative of good reliability.

### Variables and outcome measures

The questionnaire consisted of three sections: (1) Participant sociodemographics: This section asked about the participants' age, gender, years of professional experience, formal postgraduate training, type of continuing education, and practice setting. (2) Diagnostic knowledge of EPLs: This section asked six questions about diagnostic knowledge of the pathways of communication between the endodontic and periodontal tissues, clinical and functional signs, criteria for determining mixed vs. true EPLs, relevant pathology, types of radiographs used to diagnose EPLs, and characteristic radiographic signs of EPLs. (3) Prognostic and therapeutic knowledge of EPLs: Similar to the diagnostic section, this section also had six questions, but they focused on criteria for deciding whether to retain a tooth affected by EPLs, approaches for treating the various clinical manifestations of EPLs, success rates of treatments for EPLs, and follow-up treatment plans for patients treated for EPLs. The diagnostic items were designed to assess recognition of commonly accepted clinical features and foundational diagnostic principles related to EPLs rather than to replicate the full complexity of case-based diagnostic decision-making or to directly assess the specific staging, grading, and classification components of the 2017 AAP/EFP system. Accordingly, some items were intentionally framed as concept-based questions to evaluate fundamental clinical understanding rather than higher-order clinical reasoning.

To assess participants' awareness of EPLs, each participant's knowledge score was calculated from their responses to the questionnaire. Scores on individual items were determined as follows: Each individual correct response earned participants 1 point, while each individual incorrect response earned participants 0 points. When evaluating multiple-choice questions (i.e., those with multiple correct options), each option was evaluated separately using a predetermined scoring key. Correct answers were assigned 1 point, and incorrect answers 0 points. Scores for individual multiple-choice questions were then determined by adding up the number of points associated with the participant's selections for that question. An example would be the question “When do you clinically and radiographically evaluate the healing of EPLs?” As both “3 months” and “6 months” were considered correct answers, participants who selected both options could receive combined/total points for that question. Participants could select some, but not all, correct options and still receive partial credit, whereas if they selected all the correct options for a specific item, they would receive full credit. Consequently, full knowledge regarding a particular item was defined as identifying all predefined correct response options for that item without omitting any, whereas partial knowledge was defined as identifying at least one, but not all, of the correct options for that same item.

Diagnostic, therapeutic/prognostic, and total knowledge scores were derived by combining individual item scores. A maximum possible score of 16 was obtainable for diagnostic knowledge, a maximum possible score of 14 was attainable for therapeutic/prognostic knowledge, and a maximum possible score of 30 was attainable for overall knowledge about EPLs. In order to increase transparency, the complete scoring key for all questionnaire items has been included as Supplementary Table S1. Given that there were no penalties assessed against respondents for providing an incorrect answer, it is likely that this recognition-based assessment methodology slightly overstated respondent knowledge based on their ability to guess. It should also be noted that the method of assessing respondent knowledge described herein aligns with the scoring methodology employed in the original study from which the questionnaire was developed (14).

The knowledge level of each participant was grouped into one of three categories on the basis of the percentage of the total knowledge score that the participant had achieved: good knowledge (>70%), average knowledge (50–70%), and poor knowledge (< 50%). These cutoff thresholds were defined a priori and are consistent with those commonly used in knowledge, attitude, and practice (KAP) studies, facilitating comparison with the literature rather than serving as externally validated competency benchmarks (14).

### Data analysis

The initial data analysis involved systematic data cleaning to identify and address missing values, duplicate entries, and inconsistent responses. Descriptive statistics were used to represent the distribution of the participants' sociodemographic characteristics, as well as their general level of knowledge. Pearson's chi-square test was used to examine associations between participants' sociodemographic variables and their knowledge of diagnostic, therapeutic, and prognostic procedures related to EPLs.

Fisher's exact test was used when the number of expected cell counts was less than 5 in more than 20% of cells, especially in subgroup analyses that had smaller sample sizes. Simple logistic regression models were developed to evaluate crude odds ratios (CORs) and the corresponding 95% confidence intervals for each of the predictor variables for good knowledge in both the diagnostic and therapeutic areas. Variables that demonstrated a *p*-value of less than 0.25 in the univariate analysis were then entered into a multivariable logistic regression model to identify independent predictors of good knowledge and to generate adjusted odds ratios (AORs).

In addition, during the development of the multivariable logistic regression model, some of the reference categories for selected predictor variables were redefined to improve the interpretability of the AOR after adjusting for other potential confounding variables. Thus, the differences between COR and AOR are due to the adjustment for other variables rather than an error in the analysis. The likelihood ratio (LR) method, both forward and backward, was evaluated initially to determine the stability of the model. The multivariable model was developed via the “enter” method based on theoretical relevance and statistical contribution to the model. Because age and years of professional experience are conceptually related variables, potential multicollinearity between these predictors was assessed during model development (e.g., by examining correlation and model stability). Both variables were retained in the final model based on their theoretical relevance and acceptable contribution to model interpretability.

All statistical analyses were performed via IBM SPSS Statistics for Windows, Version 27.0 (IBM Corporation, Armonk, New York, USA), with a *p*-value less than 0.05 considered statistically significant. This study was reported in accordance with the STROBE (Strengthening the Reporting of Observational Studies in Epidemiology) guidelines for cross-sectional studies. The checklist is available as Supplementary Table S2.

## Results

Table 1 presents the sociodemographic characteristics of the study participants. A total of 380 respondents, consisting of females (35.3%) and males (64.7%), participated in the study. Most of the participants were between the ages of 25 and 30 (51.3%), worked in private organizations (65.0%), and had a specialty in operative dentistry and endodontics (47.1%). Approximately half of the participants had 0–5 years of professional experience (51.7%). Additionally, the majority of the participants had a postgraduate degree (67.1%) and were engaged in continuing professional training (69.7%).

**Table 1:** Sociodemographic characteristics.

Variable	Categories	*n* (%)
Gender (*n* = 380)		
	Female	134 (35.3)
	Male	246 (64.7)
Age (*n* = 380)		
	25–30	195 (51.3)
	31–35	67 (17.6)
	36–40	75 (19.7)
	41–45	18 (4.7)
	46–50	25 (6.6)
Organization (*n* = 380)		
	Government	86 (22.6)
	Private	247 (65.0)
	Semi-government	24 (6.3)
	Others	23 (6.1)
Professional experience (*n* = 380)		
	0–5	196 (51.7)
	6–10	66 (17.4)
	11–15	83 (21.9)
	16–20	16 (4.2)
	>20	18 (4.7)
Postgraduate (*n* = 380)		
	No	125 (32.9)
	Yes	255 (67.1)
Speciality (*n* = 380)		
	Operative dentist & endodontics	179 (47.1)
	Maxillofacial	9 (2.4)
	Oral pathologist	9 (2.4)
	Orthodontist	38 (10.0)
	Periodontics	51 (13.4)
	Prosthodontist	36 (9.5)
	None	58 (15.3)
Continuing training (*n* = 380)		
	No	115 (30.3)
	Yes	265 (69.7)

Table 2 presents practitioners' knowledge of the diagnostic approach to EPLs on the basis of their responses to individual questionnaire items. Practitioners achieved the highest overall correct score for the item “What types of radiography do you carry out?” (overall correct score: 76.5%), with most respondents correctly selecting periapical radiographs (94.4%). In contrast, the lowest overall correct score was observed for the item “What type of periodontal probing allows a true EPL to be characterized?” (overall correct score: 39.4%), as more than half of the respondents incorrectly selected circumferential probing in a “V” configuration (66.3%), which was not considered correct according to the scoring key.

**Table 2:** Practitioner's knowledge of the diagnostic approach to treating EPLs based on each response.

Parameter	Options	No F (%)	Yes F (%)	Overall %
What are the causes of development of endodontic or periodontal lesion?	Apical foramen	34 (8.9)	346 (91.1)	68.3%
	Lateral root canal	66 (17.6)	308 (82.4)	
	Root perforation	78 (20.5)	302 (79.5)	
	Periodontal pocket	69 (18.2)	311 (81.8)	
	Dentinal tubule	172 (45.3)	208 (54.7)	
	Endodontic access cavity	174 (45.8)	206 (54.2)	
	Root resorption	162 (42.6)	218 (57.4)	
What are the signs to consider regarding the occurrence of EPLs?	Pulp necrosis	102 (26.8)	278 (73.2)	63.4%
	Swelling	91 (23.9)	289 (76.1)	
	Abscess	66 (17.4)	346 (91.1)	
	Pain	34 (8.9)	346 (91.1)	
	Tooth mobility with deep periodontal pocket	34 (8.9)	346 (91.1)	
	Negative pulp vitality test	104 (27.4)	275 (72.6)	
	Food impaction	122 (32.1)	258 (67.9)	
	Coronal fracture	236 (62.1)	144 (37.9)	
What type of periodontal probing allows a true EPLs to be characterized?	Circumferential as a “V”	128 (33.7)	252 (66.3)	39.4%
	Circumferential as a “U”	208 (55.0)	170 (45.0)	
What pathologies allow a differential diagnosis of EPLs?	Root cracks and fractures	85 (22.4)	295 (77.6)	65.3%
	Coronal cracks or fractures	245 (64.5)	135 (35.5)	
	Retropulpitis	138 (36.3)	242 (63.7)	
	Dental anatomical anomalies	169 (44.5)	211 (55.5)	
What types of radiography do you carry out?	Periapical	21 (5.6)	357 (94.4)	76.5%
	Bitewing	216 (57.0)	163 (43.0)	
	Periapical with a GP point in the periodontal pocket or the fistula	83 (21.8)	297 (78.2)	

The scoring criteria for multi-option items are described in the methods section, and the detailed scoring key is provided in Supplementary Table S1.

Table 3 presents practitioners' knowledge of therapeutic and prognostic approaches to EPLs according to their responses to each question. The results show that practitioners scored the highest average percentage for “Is the level of healing obtained due to the following?” (96.1%), with all the respondents correctly selecting “Yes” for the option “quality of the treatment” (100%). The practitioners scored the lowest average percentage for “Is the decision to conserve or to extract a tooth afflicted by EPLs linked to the following?” (73.2%), with the majority of the practitioners correctly choosing “Yes” for the option “situation of the tooth” (95.8%).

**Table 3:** Practitioners' knowledge of therapeutic and prognostic approaches to treating EPLs based on each response.

Parameter	Options	No F (%)	Yes F (%)	Overall %
Is the decision to conserve or to extract a tooth afflicted by EPLs linked to the following?	Degree of affliction of the tooth	53 (13.9)	327 (86.1)	73.2%
	Motivation of the patient	66 (17.4)	314 (82.6)	
	Situation of the tooth	16 (4.2)	364 (95.8)	
	Reason for consultation	107 (28.2)	273 (71.8)	
Does healing of an EPL of endodontic origin require the following?	Endodontic treatment only	175 (46.1)	205 (53.9)	79.2%
	Endodontic treatment and periodontal monitoring	26 (6.8)	354 (93.2)	
	Periodontal treatment only	344 (90.5)	36 (9.5)	
Does healing of an EPLs of periodontal origin require the following?	Endodontic treatment only	296 (77.9)	84 (22.1)	74.3%
	Endodontic and periodontic treatment	78 (20.5)	302 (79.5)	
	Periodontal treatment only associated with assessment of the pulp vitality	131 (34.5)	249 (65.5)	
Does healing of an EPLs of mixed origin require the following?	Endodontic treatment only	286 (75.3)	94 (24.7)	83.8%
	Endodontic and periodontic treatment	10 (2.6)	370 (97.4)	
	Periodontal treatment only	299 (78.7)	81 (21.3)	
Is the level of healing obtained due to the following?	Quality of the treatment	0 (0)	380 (100)	96.1%
	Degree of motivation of the patient	35 (9.2)	345 (90.8)	
	Control of the risk factors	8 (2.1)	372 (97.9)	
	Accuracy of the diagnosis	16 (4.2)	364 (95.8)	
When do you clinically and radiographically evaluate the healing of EPLs?	Immediately	282 (74.2)	98 (25.8)	73.6%
	3 months	113 (29.7)	267 (70.3)	
	6 months	90 (23.7)	290 (76.3)	

Table 4 presents practitioners‘ knowledge of the diagnostic approach to EPLs, except for the item “What type of periodontal probing allows a true EPL to be characterized?” (mean = 0.79). The results indicate that the mean scores of four of the five questions on practitioners' knowledge of the diagnostic approach to EPLs were above the expected middle value.

**Table 4:** Practitioners' knowledge of the diagnostic approach for treating EPLs.

Question	*N*	Mean (SD)	Range in data	Possible range
What are the causes of development of endodontic or periodontal lesion?	374	4.75 (1.03)	2–7	0–7
What are the signs to consider regarding the occurrence of EPLs?	379	4.99 (1.09)	3–8	0–8
What type of periodontal probing allows a true EPLs to be characterized?	378	0.79 (0.84)	0–2	0–2
What pathologies allow a differential diagnosis of EPLs?	380	2.61 (0.89)	1–4	0–4
What types of radiography do you carry out?	378	2.30 (0.67)	1–3	0–3

Table 5 presents practitioners' knowledge of therapeutic and prognostic approaches to treating EPLs. The results indicate that the mean scores for all questions were above the expected middle value.

**Table 5:** Practitioners' knowledge of therapeutic and prognostic approaches to treating EPLs.

Question	*n*	Mean (SD)	Range in data	Possible range
Is the decision to conserve or to extract a tooth afflicted by EPLs linked to the following?	380	2.93 (0.71)	0–4	0–4
Does healing of an EPL of endodontic origin require the following?	380	2.38 (0.57)	0–3	0–3
Does healing of an EPL of periodontal origin require the following?	380	2.23 (0.71)	1–3	0–3
Does healing of an EPL of mixed origin require the following?	380	2.51 (0.65)	0–3	0–3
Is the level of healing obtained due to the following?	380	3.84 (0.36)	1–4	0–4
When do you clinically and radiographically evaluate the healing of EPLs?	380	2.21 (0.76)	1–3	0–3

Table 6 presents the associations between sociodemographic characteristics and participants' knowledge levels. Participant knowledge was significantly associated with age, professional experience, specialty, and continuing training (all *p* < 0.001). No statistically significant associations were observed for organization type (*p* = 0.076) or postgraduate qualification (p = 0.105) in the bivariate analysis. With respect to age, practitioners aged 41–45 years (100%) and 46–50 years (100%) demonstrated a greater proportion of good knowledge than those aged 25–30 years (55.6%), 31–35 years (64.1%), and 36–40 years (75.7%). However, these findings should be interpreted cautiously because of the smaller sample sizes in the older age categories. Similarly, for professional experience, participants with 16–20 years (100%) and more than 20 years (100%) of experience had higher proportions of good knowledge than did those with 0–5 years (51.3%), 6–10 years (75.4%), and 11–15 years (79.0%) of experience. Across specialties, higher proportions of good knowledge were observed among maxillofacial surgeons and oral pathologists, whereas orthodontists demonstrated moderate-to-high levels of good knowledge (63.1%). Interpretation of these findings is limited by the small number of participants in certain specialty groups. Operative dentistry and endodontics constituted the largest specialty group (*n* = 179; 47.1%) and represented a high proportion of good knowledge (79.8%). Other specialties also presented relatively high levels of good knowledge, including periodontology (73.4% among 51 participants) and prosthodontics (74.3% among 36 participants).

**Table 6:** Association between sociodemographic characteristics and participants' knowledge.

Variable	Categories	Poor *n* (%)	Average *n* (%)	Good *n* (%)	Chi-square (DF)	*P*
Gender	Female	0 (0)	49 (38.3)	79 (61.7)	2.43 (1)	0.254
	Male	1 (0.4)	75 (31.0)	166 (68.6)		
Age	25–30	0 (0)	84 (44.4)	105 (55.6)	46.91 (8)	**< 0.001**
	31–35	1 (1.6)	22 (34.4)	41 (64.1)		
	36–40	0 (0)	18 (24.3)	56 (75.7)		
	41–45	0 (0)	0 (0)	18 (100)		
	46–50	0 (0)	0 (0)	25 (100)		
Organization	Government	0 (0)	18 (20.9)	68 (79.1)	11.32 (6)	0.076
	Private	1 (0.4)	88 (37.1)	148 (62.4)		
	Semi-government	0 (0)	9 (37.5)	15 (62.5)		
	Others	0 (0)	9 (39.1)	14 (60.9)		
Professional experience	0–5	0 (0)	92 (48.7)	97 (51.3)	54.98 (8)	**< 0.001**
	6–10	1 (1.5)	15 (23.1)	49 (75.4)		
	11–15	0 (0)	17 (21.0)	64 (79.0)		
	16–20	0 (0)	0 (0)	16 (100)		
	>20	0 (0)	0 (0)	18 (100)		
Post-graduate	No	0 (0)	49 (40.2)	73 (59.8)	3.97 (2)	0.105
	Yes	1 (0.4)	75 (30.2)	172 (69.4)		
Specialty	Operative dentistry & endodontics	0 (0)	35 (19.2)	143 (79.8)	53.84 (14)	**< 0.001**
	Maxillofacial	0 (0)	0 (0)	9 (100)		
	Oral pathologist	0 (0)	0 (0)	9 (100)		
	Orthodontist	0 (0)	14 (36.8)	24 (63.1)		
	Periodontics	1 (2.0)	13 (26.5)	36 (73.4)		
	Prosthodontist	0 (0)	9 (25.7)	26 (74.3)		
	None	0 (0)	23 (41.8)	32 (58.2)		
Continuing training	No	0 (0)	20 (17.7)	93 (82.3)	19.89 (2)	**< 0.001**
	Yes	1 (0.4)	104 (40.5)	152 (59.1)		

Bold values indicate statistically significant results (*p* < 0.05).

Table 7 presents a multivariable logistic regression analysis performed to identify independent predictors of good knowledge regarding diagnostic and therapeutic approaches to EPLs. After adjustment for potential confounders, age group, type of organization, postgraduate qualification, and continuing professional training remained in the final model. Compared with those aged 25–30 years, practitioners aged 36–40 years were significantly more likely to demonstrate good knowledge (AOR = 2.99, 95% CI: 1.38–6.47; *p* = 0.005). Although practitioners aged 31**–**35 years presented numerically greater odds of good knowledge, this association did not reach statistical significance (AOR = 1.95, 95% CI: 0.90–4.22; *p* = 0.091). With respect to the workplace setting, practitioners working in private organizations were significantly less likely to exhibit good knowledge than those employed in government institutions (AOR = 0.38, 95% CI: 0.19–0.76; *p* = 0.001). No statistically significant associations were observed for practitioners working in semi-government or other sectors after adjustment. Compared with those without postgraduate training, those with postgraduate qualifications were more than three times as likely to demonstrate good knowledge (AOR = 3.23, 95% CI: 1.65–6.32; *p* = 0.001). Although the bivariate analysis suggested a greater proportion of good knowledge among practitioners who reported no continuing professional training (Table 6), this relationship reversed after adjustment for confounding variables. In the multivariable model, practitioners who had received continuing professional training demonstrated significantly higher odds of good knowledge (AOR = 4.55, 95% CI: 2.30–9.00; *p* < 0.001), indicating that age, professional experience, and practice setting substantially influenced the unadjusted association.

**Table 7:** Factors associated with the participants' knowledge via simple and multiple logistic regression analysis.

Variable	Categories	COR	*P*	AOR	*P*
Gender	Female	1			
	Male	1.36 (0.87, 2.11)	0.184		
Age	25–30	1		1	
	31–35	1.43 (0.79, 2.56)	0.235	1.95 (0.90, 4.22)	0.091
	36–40	2.49 (1.36, 4.55)	0.003	2.99 (1.38, 6.47)	0.005
	41–45	–	–	–	–
	46–50	–	–	–	–
Organization	Government	1		1	
	Private	0.44 (0.25, 0.79)	0.006	0.38 (0.19, 0.76)	0.001
	Semi–government	0.44 (0.17, 1.17)	0.100	0.62 (0.20, 1.92)	0.412
	Others	0.41 (0.15, 1.10)	0.078	0.49 (0.15, 1.56)	0.229
Professional experience	0–5	1			
	6–10	2.91 (1.54, 5.47)	0.001	–	–
	11–15	3.57 (1.95, 6.55)	< 0.001	–	–
	16–20	–	–	–	–
	>20	–	–	–	–
Postgraduate	No	1			
	Yes	1.52 (0.97, 2.39)	0.070	3.23 (1.65, 6.32)	0.001
Specialty	Operative dentistry & endodontics	1		1	
	Maxillofacial	–		–	–
	Oral pathologist	–	–	–	–
	Orthodontist	–	–	–	–
	Periodontics	0.73 (0.38, 1.41)	0.352	–	–
	Prosthodontist	2.30 (0.99, 5.28)	0.051	–	–
	None	1.11 (0.59, 2.09)	0.758	–	–
Continuing training	No	1			
	Yes	0.31 (0.18, 0.54)	< 0.001	4.55 (2.30, 9.00)	< 0.001

The data are presented as odds ratios with 95% confidence intervals. COR, crude odds ratio; AOR, adjusted odds ratio; confidence interval. Reference categories were respecified in the multivariable logistic regression model to improve interpretability after adjustment for potential confounders; therefore, differences in the direction or magnitude of associations between crude and adjusted models reflect covariate adjustment rather than analytical inconsistency. Variables with *p* < 0.25 in univariable analyses were considered for inclusion in the multivariable model, and *p* < 0.05 was considered statistically significant. Operative dentistry and endodontics were used as the reference category for specialty in both the crude and adjusted analyses. Categories shown as “–” were not estimable due to sparse data or quasi-complete separation.

## Discussion

This study employed a cross-sectional survey among dental practitioners in Pakistan in which self-administered questionnaires were used to assess their knowledge of diagnostic and therapeutic approaches for endodontic-periodontal lesions (EPLs). Overall, the findings indicate that dental practitioners demonstrated a high level of knowledge of EPLs, with stronger performance in therapeutic and prognostic domains than in diagnostic domains.

The findings of this study should be interpreted within the broader context of contemporary periodontal classification frameworks, including the 2017 World Workshop Classification of Periodontal and Peri-Implant Diseases and Conditions (AAP/EFP), which represents the current framework for periodontal diseases, including EPLs (10, 11), while recognizing that the questionnaire used in this study primarily assessed foundational diagnostic concepts rather than specific staging, grading, or classification components of that system. Importantly, key EPL-specific classification components, such as root damage status and the distinction between EPLs in periodontitis vs. non-periodontitis patients, were not directly assessed by the questionnaire. Accordingly, the present instrument should not be interpreted as a direct assessment of knowledge of the full 2017 World Workshop classification. Variability in clinicians' diagnostic responses observed in this study may partly reflect differences in familiarity with updated classification systems and terminology, including those introduced in the 2017 World Workshop classification (10).

The study revealed that dental practitioners were able to provide the most accurate answers to questions concerning radiographic assessments, with an average correct response rate of 76.5%. Dental practitioners also had high levels of agreement when asked to identify periapical radiographs (94.4%) and radiographs with a gutta-percha point placed in the periodontal pocket (78.2%). The results support prior findings regarding radiographic assessment in dental practice, such as those of a study in Burkina Faso, where 91% of dental surgeons used diagnostic periapical radiographs with a gutta-percha point located in the periodontal pocket or fistula ostium (14). These findings indicate that dental practitioners demonstrated a reasonable understanding of adjunctive radiographic methods for differentiating between periodontal and endodontic etiologies (15).

With respect to therapeutic and prognostic knowledge of EPLs, dental practitioners presented the greatest level of comprehension regarding healing processes, with an average correct response rate of 96.1%. Many respondents agreed that the quality of treatment (100%) and control of risk factors (97.9%) were important in facilitating healing. Research has recently emphasized the role of patient motivation in the selection of treatments for periodontitis; these motivations include anticipated aesthetic outcomes, costs, length of time required for completion, and durability of the selected treatment (16). Prior studies have reported that 24% of practitioners perform endodontic treatments independently; however, it has been noted that purulent discharge from periodontal pockets usually ceases spontaneously after successful endodontic treatment in acute cases (15, 17, 18). Periodontal signs are known to disappear after endodontic treatment (19–22).

In general, the findings of this study indicated that dental practitioners' knowledge of diagnostic and therapeutic approaches to EPLs was above average, with mean scores greater than the midpoint of the scale. The questionnaire was constructed to measure the respondents' ability to recognize principles of diagnosis but was not intended to measure the level of higher-order clinical reasoning that would be required by a clinician. Also, many of the items in this survey were specifically designed to elicit answers that directly relate to specific concepts (e.g., what are the determinants of healing?) that most clinicians will understand. This could have resulted in very consistent responses across all items and may limit the evaluation of how well respondents can apply their clinical reasoning skills to patient-specific situations. As in other studies, these findings suggest that there is a positive relationship between knowledge and skill in EPLs management, with approximately 70% of dental practitioners possessing above-average diagnostic knowledge and nearly all (up to 98%) possessing above-average therapeutic and prognostic knowledge (14). The findings suggest a significant difference between therapeutic knowledge and diagnostic knowledge; therefore, educational interventions targeting diagnostic accuracy would be beneficial for strengthening both diagnostic and therapeutic abilities in the management of EPLs (23). Determining accurate diagnosis and making informed treatment decisions are two key components in determining whether therapeutic interventions will be effective; thus, developing diagnostic competency should occur concurrently with developing therapeutic competence in the management of EPLs (23).

In this study, practitioners showed strong evidence of possessing knowledge about therapeutic approaches relative to diagnostic procedures for EPLs. This indicates that there is a lack of balance between practitioners' treatment competence and diagnostic accuracy in regard to EPLs management. The fact that practitioners have poor diagnostic skills in clinical assessments, communication pathways, and radiographic interpretations reinforces the need for ongoing education/training to improve diagnostic skills. Improved collaboration between operative dentists, endodontists, and periodontists can lead to more accurate diagnoses and improved treatment plans, which can lead to better patient care. Bivariate analyses revealed a positive association between practitioners' knowledge of EPLs and sociodemographic factors such as age, years of professional practice, specialty, and continuing professional training. The organization type and postgraduate qualifications were not shown to be positively associated with knowledge of EPLs via bivariate analysis. Older age groups and greater numbers of years of professional practice had higher proportions of good knowledge; however, owing to smaller subgroup sizes, caution should be taken when interpreting these results.

Among the different specialties, operative dentistry and endodontics constituted the most represented specialty group (47.1%) in the survey and represented a high proportion of good knowledge (79.8%). Practitioners in operative dentistry and endodontics also demonstrated one of the highest levels of knowledge among other specialties, such as periodontology (73.4%), prosthodontics (74.3%), and orthodontics (63.1%). These findings should be interpreted with caution, as the relatively small number of participants within certain specialty groups may limit the robustness of subgroup comparisons. Previous research exploring the relationship between practitioners' knowledge of EPL diagnosis and treatment and demographic characteristics remains limited. Overall, the results suggest variability in knowledge across dental disciplines, indicating both the presence of broadly adequate understanding and notable disparities that may influence clinical decision-making.

Furthermore, age and professional training-related factors (i.e., postgraduate qualifications and continuing professional development) are important predictors of knowledge of EPLs, and older practitioners, as well as those who engage in formalized training pathways, are more likely to possess knowledge of EPLs. Older practitioners may have a larger body of clinical experience to draw upon when managing complex cases (such as EPLs); similarly, continued professional development supports the maintenance and growth of clinical expertise (24–26).

Although numerous international studies have assessed practitioners‘ knowledge of EPLs, few national-level studies have been conducted in Pakistan, and fewer still have utilized multivariable regression analyses to assess the independent effects of demographic and professional factors on knowledge gaps (14, 27). Therefore, the current results will assist in filling this gap and provide a nationally representative view of practitioners' knowledge and the factors associated with this knowledge. Although the results may be useful for similar low- and middle-income countries with analogous training systems, direct generalization should be performed with caution without multi-country validation.

Despite the rapid evolution of dental care in Pakistan, dental education needs to reflect current evidence-based scientific developments (13). In Pakistan, endodontics is part of the operative dentistry specialty, referred to as operative dentistry and endodontics. Specialty training is provided primarily through the FCPS residency program offered by the College of Physicians and Surgeons of Pakistan (CPSP) or the MDS university postgraduate pathway. Clinicians in this field dedicate more than 70% of their time to endodontics, and similarly, more than 70% of the specialty curriculum focuses on managing pulp and peri-radicular diseases (28).

This study has several limitations that should be taken into consideration. The first limitation is that the use of self-administered questionnaires may introduce respondent bias, i.e., respondents may overstate their level of knowledge or respond in a manner they perceive as being socially acceptable. Furthermore, because it was not possible to request clarification from respondents when completing the questionnaire, some respondents may have provided answers to questions that they did not understand correctly, thereby introducing errors in the collected data. A second limitation is that the non-probability snowball sampling method used in this study introduces selection bias, since respondents were requested to forward the survey to their colleagues. Therefore, the sample obtained in this study may not fully represent the larger population of dental practitioners in Pakistan, thus limiting the generalizability of the findings. An additional limitation is that age and years of professional experience are conceptually related variables and may introduce multicollinearity. Although this was assessed during model development, some degree of residual overlap may remain; therefore, the independent effects of these variables should be interpreted with caution. Another major limitation is that the survey was based on the assessment of recognition-based or factual knowledge and therefore did not assess clinical decision-making using case-based formats. The scoring strategy used for multiple-choice items provided no penalty for incorrect responses (i.e., guessing) and, therefore, likely resulted in a small overestimation of knowledge due to guessing. In addition, some of the items represented a simplification of the complex nature of actual clinical diagnostic and treatment decisions in EPLs. Furthermore, many of the items were designed as simple knowledge items rather than scenario-based assessments; therefore, they may have failed to evaluate higher-order clinical reasoning processes. As such, these results are best viewed as representing trends in general knowledge rather than defining overall clinical competency.

Nonetheless, the findings of the study provide an essential source of information regarding the areas of strength in terms of dental practitioners' knowledge of diagnosing and treating EPLs, as well as the areas where there are knowledge deficits that could be addressed through targeted educational efforts. This study provides an opportunity for developing evidence-based clinical guidelines for the diagnosis and treatment of EPLs, particularly within the context of Pakistan. The study also suggests a need to develop and implement continuing professional development programs specifically designed for various specialty areas and varying levels of professional experience. The provision of structured training opportunities allows dental practitioners to remain knowledgeable about the most recent diagnostic methods and treatment protocols available to them, which in turn enhances the quality of care they provide to their patients. Future studies should investigate the effectiveness of such training programs and assess whether the implementation of standardized clinical guidelines improves treatment outcomes.

To address the knowledge deficits identified in this study, we suggest the development of focused continuing education programs that meet the unique needs of dental practitioners. We also propose the establishment of standard clinical guidelines for the diagnosis and treatment of EPLs to ensure consistent treatment protocols across all clinics. Mentorship programs between more senior dental practitioners and less experienced practitioners will facilitate the dissemination of knowledge and improvement in clinical skills among less experienced practitioners. By combining these strategies, diagnostic accuracy, treatment consistency, and ultimately, patient outcomes can be improved in the management of EPLs.

## Conclusions

Endodontic–periodontal lesions present ongoing challenges in diagnosis and management, requiring sound clinical judgment and interdisciplinary coordination. The findings of this study indicate that dental practitioners in Pakistan generally possess adequate therapeutic knowledge, whereas diagnostic understanding remains relatively moderate. The multivariable analysis revealed that age (36**–**40 years), postgraduate qualifications, continuing professional training, and government-sector employment were significant predictors of good knowledge. These results underscore the importance of structured postgraduate education and continuous professional development programs to increase diagnostic accuracy and treatment consistency for endodontic–periodontal lesions.

## Data Availability

The original contributions presented in the study are included in the article/Supplementary material, further inquiries can be directed to the corresponding authors.
