# Supplementary material for: Variability in diagnostic and therapeutic decision-making for endodontic-periodontal lesions: evidence from a cross-sectional study
Source: Front Public Health. 2026 Apr 30;14:1795184. doi: 10.3389/fpubh.2026.1795184 (PMC13171735; doi:10.3389/fpubh.2026.1795184)
Supplement: Supplementary file 1 [file Table_1.docx]

**Supplementary Table S1. Scoring key for questionnaire items assessing knowledge of endodontic-periodontal lesions (EPLs)**

For each response option, the correct answer was predefined as either “Yes” or “No.” A score of 1 was assigned for a correct response and 0 for an incorrect response. For items with multiple response options, the score for each item was calculated as the sum of correctly answered options. Incorrect selections were not negatively penalized; therefore, participants selecting some, but not all, correct options received partial credit. For the healing follow-up item, both “3 months” and “6 months” were considered correct responses, and selecting both resulted in cumulative scoring. This supplementary table is intended to improve the transparency of the scoring algorithm used for questionnaire-based knowledge assessment. Because incorrect selections were not negatively penalized, this recognition-based scoring approach may modestly overestimate knowledge due to guessing.

| **Domain** | **Question** | **Response option** | **Correct response** | **Score** |
| --- | --- | --- | --- | --- |
| **Diagnostic knowledge** | What are the causes of development of endodontic or periodontal lesion? | Apical foramen | Yes | 1 |
|  | What are the causes of development of endodontic or periodontal lesion? | Lateral root canal | Yes | 1 |
|  | What are the causes of development of endodontic or periodontal lesion? | Root perforation | Yes | 1 |
|  | What are the causes of development of endodontic or periodontal lesion? | Periodontal pocket | Yes | 1 |
|  | What are the causes of development of endodontic or periodontal lesion? | Dentinal tubule | Yes | 1 |
|  | What are the causes of development of endodontic or periodontal lesion? | Endodontic access cavity | No | 0 |
|  | What are the causes of development of endodontic or periodontal lesion? | Root resorption | No | 0 |
|  | What are the signs to consider regarding the occurrence of EPLs? | Pulp necrosis | Yes | 1 |
|  | What are the signs to consider regarding the occurrence of EPLs? | Swelling | Yes | 1 |
|  | What are the signs to consider regarding the occurrence of EPLs? | Abscess | Yes | 1 |
|  | What are the signs to consider regarding the occurrence of EPLs? | Pain | No | 0 |
|  | What are the signs to consider regarding the occurrence of EPLs? | Tooth mobility with deep periodontal pocket | Yes | 1 |
|  | What are the signs to consider regarding the occurrence of EPLs? | Negative pulp vitality test | Yes | 1 |
|  | What are the signs to consider regarding the occurrence of EPLs? | Food impaction | No | 0 |
|  | What are the signs to consider regarding the occurrence of EPLs? | Coronal fracture | No | 0 |
|  | What type of periodontal probing allows a true EPLs to be characterized? | Circumferential as a "V" | No | 0 |
|  | What type of periodontal probing allows a true EPLs to be characterized? | Circumferential as a "U" | Yes | 1 |
|  | What pathologies allow a differential diagnosis of EPLs? | Root cracks and fractures | Yes | 1 |
|  | What pathologies allow a differential diagnosis of EPLs? | Coronal cracks or fractures | No | 0 |
|  | What pathologies allow a differential diagnosis of EPLs? | Retropulpitis | Yes | 1 |
|  | What pathologies allow a differential diagnosis of EPLs? | Dental anatomical anomalies | Yes | 1 |
|  | What types of radiography do you carry out? | Periapical | Yes | 1 |
|  | What types of radiography do you carry out? | Bitewing | No | 0 |
|  | What types of radiography do you carry out? | Periapical with a GP point in the periodontal pocket or the fistula | Yes | 1 |
| Therapeutic/prognostic knowledge | Is the decision to conserve or to extract a tooth afflicted by EPLs linked to the following? | Degree of affliction of the tooth | Yes | 1 |
|  | Is the decision to conserve or to extract a tooth afflicted by EPLs linked to the following? | Motivation of the patient | Yes | 1 |
|  | Is the decision to conserve or to extract a tooth afflicted by EPLs linked to the following? | Situation of the tooth | Yes | 1 |
|  | Is the decision to conserve or to extract a tooth afflicted by EPLs linked to the following? | Reason for consultation | No | 0 |
|  | Does healing of an EPLs of endodontic origin require the following? | Endodontic treatment only | Yes | 1 |
|  | Does healing of an EPLs of endodontic origin require the following? | Endodontic treatment and periodontal monitoring | Yes | 1 |
|  | Does healing of an EPLs of endodontic origin require the following? | Periodontal treatment only | No | 0 |
|  | Does healing of an EPLs of periodontal origin require  the following? | Endodontic treatment only | No | 0 |
|  | Does healing of an EPLs of periodontal origin require the following? | Endodontic and periodontic treatment | Yes | 1 |
|  | Does healing of an EPLs of periodontal origin require the following? | Periodontal treatment only associated with assessment of the pulp vitality | Yes | 1 |
|  | Does healing of an EPL of mixed origin require the following? | Endodontic treatment only | No | 0 |
|  | Does healing of an EPL of mixed origin require the following? | Endodontic and periodontic treatment | Yes | 1 |
|  | Does healing of an EPL of mixed origin require the following? | Periodontal treatment only | No | 0 |
|  | Is the level of healing obtained due to the following? | Quality of the treatment | Yes | 1 |
|  | Is the level of healing obtained due to the following? | Degree of motivation of the patient | Yes | 1 |
|  | Is the level of healing obtained due to the following? | Control of the risk factors | Yes | 1 |
|  | Is the level of healing obtained due to the following? | Accuracy of the diagnosis | Yes | 1 |
|  | When do you clinically and radiographically evaluate the healing of EPLs? | Immediately | No | 0 |
|  | When do you clinically and radiographically evaluate the healing of EPLs? | 3 months | Yes | 1 |
|  | When do you clinically and radiographically evaluate the healing of EPLs? | 6 months | Yes | 1 |

**Note:** This supplementary table is intended to improve transparency of the scoring algorithm used for questionnaire-based knowledge assessment.
