# Supplementary material for: Variability in diagnostic and therapeutic decision-making for endodontic-periodontal lesions: evidence from a cross-sectional study
Source: Front Public Health. 2026 Apr 30;14:1795184. doi: 10.3389/fpubh.2026.1795184 (PMC13171735; doi:10.3389/fpubh.2026.1795184)
Supplement: Supplementary file 2 [file Table_2.docx]

**STROBE Checklist for “Variability in Diagnostic and Therapeutic Decision-Making for Endo-Periodontal Lesions: Evidence from a Cross-Sectional Study”**

| Item No | Recommendation | Respected? | Comments / Quotes |
| --- | --- | --- | --- |
| 1(a) | Indicate the study’s design with a commonly used term in the title or abstract | Yes | The title includes “Cross-Sectional Study,” and the abstract states that a cross-sectional survey was conducted among dental practitioners. |
| 1(b) | Provide an informative and balanced summary of what was done and found | Yes | The abstract summarizes the background, cross-sectional survey design, questionnaire-based methodology, participant characteristics, main findings, and conclusion. |
| 2 | Explain the scientific background and rationale | Yes | The Introduction explains the clinical complexity of endodontic-periodontal lesions, the challenges in diagnosis and treatment, and the need to evaluate practitioners’ knowledge. |
| 3 | State specific objectives | Yes | The study objective is clearly stated: to evaluate the diagnostic, therapeutic, and prognostic approaches to endodontic-periodontal lesions among dental practitioners and identify predictors of knowledge variability. |
| 4 | Present key elements of study design early | Yes | The Methods identify the study as an observational descriptive cross-sectional study conducted among registered dental practitioners in Pakistan. |
| 5 | Describe setting, location, and relevant dates | Yes | The study setting, country, and data collection period are reported: Pakistan; January 2024 to March 2024; questionnaire distributed online. |
| 6(a) | Give the eligibility criteria, and the sources and methods of selection of participants | Yes | Eligibility criteria are defined: registered dental practitioners currently practicing in Pakistan, with BDS or equivalent, who consented and completed the questionnaire. Exclusion criteria are also specified. |
| 6(b) | For matched studies, give matching criteria and number of exposed and unexposed | N/A | Not applicable because this was a cross-sectional survey and did not involve matched groups. |
| 7 | Clearly define all outcomes, exposures, predictors, potential confounders, and effect modifiers | Yes | The questionnaire domains and outcome measures are described, including sociodemographic predictors, diagnostic knowledge, therapeutic/prognostic knowledge, and composite knowledge scores. |
| 8* | For each variable of interest, give sources of data and details of methods of assessment | Yes | Data were obtained using a self-administered questionnaire adapted from a previous study, reviewed by experts, pilot tested, and used to assess knowledge related to endodontic-periodontal lesions. |
| 9 | Describe any efforts to address potential sources of bias | Yes | The manuscript reports duplicate-response checking, exclusion of incomplete surveys, anonymization, and acknowledges selection and response bias in the limitations. |
| 10 | Explain how the study size was arrived at | Yes | Sample size was estimated using the Raosoft Calculator with 95% confidence level, 5% margin of error, and 50% response distribution; minimum required sample was 385, and 380 complete responses were analyzed. |
| 11 | Explain how quantitative variables were handled in the analyses | Yes | Knowledge scores were summed and categorized into good, average, and poor knowledge based on predefined percentage thresholds (>70%, 50–70%, <50%). |
| 12(a) | Describe all statistical methods, including those used to control for confounding | Yes | Descriptive statistics, Pearson’s chi-square test, Fisher’s exact test, simple logistic regression, and multivariable logistic regression were used. The model-building process and adjusted odds ratios are described. |
| 12(b) | Describe any methods used to examine subgroups and interactions | Yes | Subgroup comparisons were performed across age, professional experience, specialty, organization type, postgraduate status, and continuing training. |
| 12(c) | Explain how missing data were addressed | Yes | Incomplete responses were omitted from the analysis; duplicates and incomplete entries were removed during data cleaning. |
| 12(d) | If applicable, describe analytical methods taking account of sampling strategy | Yes | The manuscript states that a nonprobability snowball sampling method was used and findings from subgroup analyses with small cell sizes should be interpreted with caution. |
| 12(e) | Describe any sensitivity analyses | N/A | Sensitivity analyses were not reported and were not required for the descriptive cross-sectional design. |
| 13(a) | Report numbers of individuals at each stage of study | Yes | The manuscript reports that 391 responses were received, 11 were omitted due to incompleteness or duplication, and 380 were included in the final analysis. |
| 13(b) | Give reasons for non-participation at each stage | Yes | Reasons for exclusion are reported: incomplete responses and duplicate submissions. |
| 13(c) | Consider use of a flow diagram | N/A | A flow diagram was not included; this is optional for survey-based cross-sectional studies. |
| 14(a) | Give characteristics of study participants | Yes | Table 1 presents participant characteristics including gender, age, organization, professional experience, postgraduate status, specialty, and continuing training. |
| 14(b) | Indicate number of participants with missing data for each variable of interest | Partly | The manuscript explains that incomplete surveys were excluded, but variable-specific missing data are not separately tabulated for every item. |
| 15 | Report numbers of outcome events or summary measures | Yes | Tables 2–7 summarize diagnostic, therapeutic, and prognostic knowledge items, mean scores, associations, and regression outcomes. |
| 16(a) | Give unadjusted estimates and, if applicable, adjusted estimates with precision | Yes | Table 7 reports crude odds ratios (COR), adjusted odds ratios (AOR), 95% confidence intervals, and p-values. |
| 16(b) | Report category boundaries when continuous variables were categorized | Yes | Age, professional experience, and knowledge-score thresholds are clearly categorized and defined in the Methods and Results. |
| 16(c) | If relevant, consider translating estimates of relative risk into absolute risk | N/A | Not applicable because logistic regression and odds ratios were used rather than risk estimates. |
| 17 | Report other analyses done, e.g., subgroups and interactions | Yes | The manuscript reports subgroup analyses by sociodemographic and professional variables and discusses multivariable logistic regression findings. |
| 18 | Summarise key results with reference to study objectives | Yes | The Discussion opens by summarizing the main findings in relation to practitioners’ diagnostic and therapeutic knowledge of endodontic-periodontal lesions. |
| 19 | Discuss limitations of the study | Yes | The Discussion acknowledges limitations including self-reported data, response bias, inability to clarify responses, snowball sampling, limited generalizability, and overlap between age and professional experience. |
| 20 | Give a cautious overall interpretation of results considering objectives, limitations, multiplicity of analyses, results from similar studies, and other relevant evidence | Yes | The findings are interpreted cautiously in relation to prior studies, clinical implications, educational needs, and the limitations of the study design. |
| 21 | Discuss the generalisability (external validity) of the study results | Yes | The Discussion notes that the findings may inform similar low- and middle-income settings but advises caution regarding direct generalization without multicountry validation. |
| 22 | Give the source of funding and the role of the funders | Yes | The manuscript states that the work was funded by the Deanship of Graduate Studies and Scientific Research at Jouf University under grant No. DGSSR-2025-01-01127. |
